# Teaching Strategies in Psychiatric Nursing Based on Bloom’s Taxonomy of Educational Objectives

**DOI:** 10.7759/cureus.57759

**Published:** 2024-04-07

**Authors:** Zheng Jia, Jesse M Balinas

**Affiliations:** 1 Educational Management, Angeles University Foundation, Angeles, PHL

**Keywords:** psychiatric nursing, bloom’s taxonomy of educational objective, teaching strategies, bloom’s taxonomy, psychiatric nursing education

## Abstract

Bloom’s Taxonomy of Educational Objective (BTEO), as a classic method for categorizing educational objectives, provides a clear and specific framework for formulating teaching goals in the global education field. Psychiatric nursing, as a highly specialized discipline, demands high requirements for students in both theoretical knowledge and practical skills. However, traditional teaching methods often focus excessively on knowledge impartation, neglecting the cultivation of students’ practical abilities and emotional attitudes. Therefore, the purpose of this study was to Improve the existing psychiatric nursing curriculum by combining Bloom's educational goals theory, which emphasizes the comprehensive development of cognitive, affective, and psychomotor skills, providing new perspectives and methods for psychiatric nursing education. By applying BTEO, specific teaching goals at different levels can be defined more clearly, and corresponding teaching strategies and methods can be employed to achieve these goals. Furthermore, the application of BTEO requires effective interaction between teachers and students. Teachers need to monitor students’ learning progress, adjust teaching strategies promptly, and ensure that students comprehensively grasp knowledge and skills. Thus, the application of this teaching strategy contributes to improving the quality of psychiatric nursing education and cultivating outstanding psychiatric nursing professionals. We hope to cultivate students' theoretical knowledge and practical skills in mental care by helping them develop their overall skills.

## Introduction

The origin of Bloom’s Taxonomy of Educational Objectives (BTEO) can be traced back to the 1950s during the educational reform movement in the United States. At that time, dissatisfaction with traditional teaching methods arose in the United States education sector, with a belief that these methods overly emphasized memorization and standardized testing, neglecting the cultivation of students’ thinking and innovation abilities [1,2]. Concurrently, societal expectations for education were continuously rising, aiming for education to better meet the individual and societal developmental needs of students.

In this context, Benjamin Bloom and his colleagues dedicated themselves to developing a new set of educational objectives and a classification method. They argued that to enhance students’ thinking abilities, it was crucial to clearly define educational goals and categorize them. Therefore, based on the study and understanding of human cognitive processes, they proposed an educational objective classification method that encompassed cognitive, affective, and psychomotor domains. The cognitive domain’s hierarchical levels range from lower to higher, including knowledge, comprehension, application, analysis, synthesis, and evaluation[1]. These levels focus on students’ varying levels of understanding and application of knowledge, progressing from recalling facts to making value judgments and evaluations at the highest level. The affective domain’s levels focus on students’ emotional experiences and the formation of values, starting from acceptance and gradually developing into personalized levels within a value system [3].

The goals of the psychomotor domain are to help students master necessary skills and actions, enabling them to apply them proficiently in practical situations. This classification method gained widespread promotion and application in the United States, exerting a profound global impact not only on education but also on other fields such as psychology, training, and human resources development [4,5]. The purpose of this study was to integrate Bloom's educational goal theory into psychiatric nursing education and explore how to cultivate nursing students into psychiatric nurses who meet clinical needs in the areas of knowledge, emotion, and motor skills.

## Technical report

The classification of educational objectives covering cognitive, emotional, and psychomotor fields is shown in Table 1.

**Table 1:** Bloom’s Taxonomy of Educational Objective

Domain	Hierarchy	Describe
Cognitive Domain	Knowledge	Memorize and recall facts, concepts, and information
	Comprehension	Understand the fundamental meaning and concepts of the learned content
	Application	Apply acquired knowledge to new situations or problem-solving
	Analysis	Break down knowledge into its constituent parts and understand their inherent relationships
	Synthesis	Combine various parts to form a new whole or solution
	Evaluation	Evaluate the value of knowledge or information based on specific standards or criteria
Affective Domain	Receive	Students are willing to encounter or pay attention to certain concepts or phenomena.
	Reflect	Students develop positive emotional responses or interest in the learned content.
	Form Values	Students begin to form their own values or beliefs.
	Organize Value System	Students integrate their values into a certain value system.
	Personalize Value System	Students’ values become incorporated into their lives and behaviors, becoming a part of their personality.
Psychomotor Domain	Varies by discipline	Students acquire skills and actions through learning.

Application strategies of BTEO in psychiatric nursing teaching

In psychiatric nursing, developing practical operational abilities is crucial for effectively managing patient care. By categorizing these abilities according to BTEO, we can ensure a comprehensive and structured approach to learning and skill development.

Categorization of Abilities

These abilities are categorized accordingly as follows.

Remembering: This includes recalling the signs and symptoms of common psychiatric disorders and memorizing the basic principles of patient confidentiality and privacy

Understanding: This includes explaining the therapeutic effects and potential side effects of commonly used psychiatric medications and interpreting patients' verbal and non-verbal communication cues

Applying: This includes applying de-escalation techniques to manage patients exhibiting aggressive behavior and utilizing therapeutic communication skills in patient interactions

Analyzing: This includes analyzing case studies to identify the underlying psychiatric conditions and differentiating between symptoms of various psychiatric disorders

Evaluating: This includes assessing the effectiveness of nursing interventions in improving patient outcomes and evaluating the appropriateness of care plans based on patient progress

Creating: This includes developing individualized care plans tailored to meet the unique needs of psychiatric patients and innovating new strategies for engaging patients in therapeutic activities [6].

By targeting these specific operational abilities within the framework of BTEO, psychiatric nursing education can equip nurses with the necessary skills to provide high-quality, patient-centered care in a mental health setting. This structured approach not only enhances learning but also ensures that nurses are prepared to address the complex needs of psychiatric patients effectively.

Clarifying Educational Goals

In psychiatric nursing education, the formulation of educational goals is a crucial step [7]. Following BTEO, teachers need to establish clear educational goals based on students’ actual situations and subject requirements, assisting students in mastering the necessary knowledge and skills [8-10]. Firstly, in the cognitive domain, educational goals aim to cultivate students’ thinking abilities and problem-solving skills [11]. In psychiatric nursing, students need to grasp basic concepts, theories, and methods, understanding and applying relevant knowledge. Teachers can use methods such as lectures, case analyses, and group discussions to help students gain an in-depth understanding of the psychiatric nursing knowledge system, fostering critical thinking and problem-solving skills. Secondly, in the affective domain, educational goals aim to cultivate students’ emotional attitudes and values [12]. In psychiatric nursing, students need to possess attitudes of empathy, care, and respect, understanding and empathizing with patients’ needs and feelings. Teachers can employ methods like role-playing [13], simulation training [14], and practical operations to allow students to experience patients’ emotions and needs firsthand [15], developing their emotional attitudes and values. Thirdly, in the psychomotor domain, educational goals aim to develop students’ practical operational abilities and technical proficiency. In psychiatric nursing, students need to master various nursing skills and methods, applying them proficiently in practical situations. Teachers can use methods such as practical operations, simulation training, and clinical internships to help students practice and master various nursing skills and methods, enhancing their technical proficiency and practical operational abilities. By clarifying educational goals, teachers can better guide students’ learning and development, cultivating psychiatric nursing professionals with good comprehensive qualities and practical abilities.

Enhancing Teaching Methods

The learning of theoretical knowledge is the first and most important stage of all achievable learning. It is crucial to help students establish learning habits and learn the ability to think independently. Therefore, we should use case discussion methods to increase students' interest in learning and independent thinking ability, group discussion methods to stimulate students' interest in learning, and role-playing methods to help students achieve empathy with patients

In psychiatric nursing education, the improvement of teaching methods is indispensable. BTEO emphasizes students’ active learning, advocating the use of various effective teaching methods, and providing teachers with new insights and directions [1]. To assist students in reaching higher cognitive levels, teachers need to continuously explore and innovate teaching methods, as illustrated in Table 2. Firstly, case analysis is an exceptionally effective teaching method. By introducing authentic patient cases, teachers can guide students in analyzing the characteristics and issues within the cases, encouraging them to think and solve problems in specific contexts. This not only helps students better understand theoretical knowledge but also cultivates their clinical thinking and problem-solving abilities. Secondly, group discussions are also a method worth promoting. By grouping students for discussions, teachers can stimulate their interest and initiative in learning, promoting communication and cooperation. In group discussions, students can freely express their opinions, share experiences, and think about problems from different perspectives, thus fostering critical thinking and a spirit of cooperation[11]. Thirdly, role-playing is a vivid teaching method. By having students simulate different roles, teachers can allow them to experience the emotions and needs of patients firsthand, cultivating empathy and a caring attitude. Additionally, role-playing helps students better understand professional roles and responsibilities, enhancing their professional qualities and practical abilities. Beyond the mentioned teaching methods, teachers can explore more effective methods based on the actual situation. For example, teachers can utilize information technology for online teaching, multimedia teaching, etc., providing students with a more diverse range of learning resources and means, enhancing their learning effectiveness and comprehensive qualities.

**Table 2:** Characteristics and Applicable Scope of Teaching Methods

Domain	Teaching methods	Characteristics	Applicability
Cognitive Domain	Case analysis	Simulates real-life situations to help students understand theoretical knowledge and cultivate clinical thinking and problem-solving skills.	Suitable for theoretical learning and problem-solving.
Affective Domain	Group discussion	Facilitates communication and collaboration, stimulates learning interest and initiative, and nurtures critical thinking and teamwork.	Applicable to independent learning, communication and collaboration, and expanding knowledge.
	Role-playing	Engaging and enjoyable, allows students to experience patients’ emotions and needs firsthand, fostering empathy and a caring attitude.	Applicable to emotional cultivation, professional ethics, and enhancing practical skills.
Psychomotor Domain	Multimedia teaching	Provides diverse learning resources and methods, expands learning time and space, and promotes personalized learning.	Suitable for diverse learning needs, independent learning, and lifelong learning.

Assessing Learning Outcomes

Assessing learning outcomes is an important aspect of the teaching process. It is helpful to understand students' learning situation, find out the problems in teaching, and provide a basis for improving teaching methods and adjusting teaching objectives. Using BTEO can help teachers evaluate students' learning outcomes comprehensively and objectively. On the one hand, teachers need to observe students' performance in teaching activities and pay attention to students' participation and contribution in group discussions, role-playing, case studies, practical exercises, and other activities. By observing students' interactions, discussions, and thought processes, teachers grasp students' ability to understand and apply psychiatric nursing. For example, the assessment of whether students actively participate in discussions, elaborate views, analyze cases, propose reasonable nursing plans, skilled use of nursing technology, and operational skills. These observations help teachers determine whether students are meeting expected learning goals. On the other hand, teachers can test students' mastery of knowledge, skills, and abilities through tests and assessments. Tests may include written tests, practical assessments, and other forms to see how well students remember and understand theoretical knowledge and how proficient they are in applying their skills in practical exercises. Evaluation can adopt self-evaluation, mutual evaluation, teacher evaluation, and other ways to encourage students to participate in the evaluation process, so as to enhance students' self-reflection and critical thinking ability. At the same time, the evaluation results can be used as the basis for improving teaching methods and adjusting teaching objectives, and help teachers to strengthen teaching activities and improve teaching effects.

## Discussion

This article integrates BTEO into the teaching of psychiatric nursing, aiming to cultivate clinical nurses with high-quality nursing skills suitable for modern clinical needs by enhancing students' cognition, learning interests, and motor skills. This teaching reform is still in its early stages in China. If successfully implemented, we plan to apply Bloom's theory to more nursing higher education courses.

Based on BTEO, we help students learn and master important knowledge and skills progressively according to the difficulty of the knowledge points, thereby deepening their understanding. Moreover, continuous feedback and adjustment play a crucial role in enhancing teaching effectiveness. By assessing students' learning outcomes, teachers can gather valuable feedback to guide students more effectively and adjust teaching methods and strategies accordingly[16-18]. To achieve this, teachers need to provide specific feedback based on assessment results. For students who have met the expected objectives, teachers should offer affirmation and encouragement to stimulate their motivation to learn. Conversely, for those who haven't met the expected objectives, teachers need to identify areas for improvement and provide constructive suggestions. Simultaneously, teachers can guide students in self-reflection, helping them identify their challenges and find methods for improvement. Additionally, teachers must promptly adjust teaching methods and strategies based on students' feedback and performance. If certain aspects of student learning outcomes are below expectations, teachers should conduct a thorough analysis of the reasons and modify teaching strategies accordingly. For instance, if students exhibit insufficient mastery of theoretical knowledge, teachers can enhance theoretical instruction by increasing classroom explanations and post-class reviews. If students' practical skills are lacking, teachers can increase the focus on practical training.

## Conclusions

In the teaching of psychiatric nursing, BTEO provides a valuable opportunity for instructors to better design courses and teaching methods, aiming to comprehensively cultivate students. In the cognitive domain, teachers can employ methods such as case analysis and group discussions to help students deepen their understanding of theoretical knowledge in psychiatric nursing. This fosters critical thinking and problem-solving skills. In the affective domain, teachers can guide students through reflection and discussions to cultivate the correct emotional attitudes and values, better preparing them for the demands of psychiatric nursing work. In the psychomotor domain, teachers can use practical exercises and simulation training to develop students’ practical skills and their ability to handle emergency situations.

Therefore, the instructional strategy in psychiatric nursing education based on BTEO is a comprehensive and systematic teaching approach. Through a holistic development of cognitive, affective, and psychomotor skills, it aids students in mastering both the theoretical knowledge and practical skills of psychiatric nursing. Simultaneously, it cultivates the right emotional attitudes and values. Over time, this approach contributes to the continuous improvement of the quality of psychiatric nursing education, nurturing a diverse pool of talents for societal development.

## References

[1] (1956). Taxonomy of Educational Objectives: The Classification of Educational Goals. https://web.archive.org/web/20201212072520id_/https://www.uky.edu/~rsand1/china2018/texts/Bloom%20et%20al%20-Taxonomy%20of%20Educational%20Objectives.pdf.

[2] Nascimento JD, Siqueira TV, Oliveira JL, Alves MG, Regino DD, Dalri MC (2021). Development of clinical competence in nursing in simulation: the perspective of Bloom's taxonomy. Rev Bras Enferm.

[3] van der Hoeven Kraft KJ, Srogi L, Husman J, Semken S, Fuhrman M (2018). Engaging students to learn through the affective domain: a new framework for teaching in the geosciences. J Geosci Educ.

[4] Verenna AA, Noble KA, Pearson HE, Miller SM (2018). Role of comprehension on performance at higher levels of Bloom's taxonomy: Findings from assessments of healthcare professional students. Anat Sci Educ.

[5] Ferris TL, Aziz SM (2005). A psychomotor skills extension to Bloom's taxonomy of education objectives for engineering education. iCEER-2005: Exploring Innovation in Education and Research.

[6] McAllister M, Happell B, Flynn T (2014). Learning essentials: what graduates of mental health nursing programmes need to know from an industry perspective. J Clin Nurs.

[7] Adams NE (2015). Bloom's taxonomy of cognitive learning objectives. J Med Libr Assoc.

[8] Krau SD (2011). Creating educational objectives for patient education using the new Bloom's Taxonomy. Nurs Clin North Am.

[9] Ramirez TV (2017). On pedagogy of personality assessment: application of Bloom's taxonomy of educational objectives. J Pers Assess.

[10] Krathwohl DR (2002). A revision of Bloom's taxonomy: an overview. Theory Pract.

[11] Bloom RS (1975). Stating educational objectives in behavioral terms. Nurs Forum.

[12] Hauer J, Quill T (2011). Educational needs assessment, development of learning objectives, and choosing a teaching approach. J Palliat Med.

[13] Fossen P, Stoeckel PR (2016). Nursing students' perceptions of a hearing voices simulation and role-play: preparation for mental health clinical practice. J Nurs Educ.

[14] Ndegwa E, Seronei A, Olubukola S (2015). Patient Simulation on First Year Nursing Degree Students [Thesis]. https://www.theseus.fi/bitstream/handle/10024/91848/Ndegwa_%20Elizabeth.pdf;jsessionid=A9998A9CB52DA473999011B636E4E667?sequence=1.

[15] Jung JH, Kim HJ, Kim JS (2020). Comparison of nursing performance competencies and practical education needs based on clinical careers of operating room nurses: a cross-sectional study. Healthcare (Basel).

[16] Brookhart SM (2017). How to Give Effective Feedback to Your Students. https://files.ascd.org/staticfiles/ascd/pdf/siteASCD/publications/books/How-to-Give-Effective-Feedback-to-Your-Students-2nd-Edition-sample-chapters.pdf.

[17] McTighe J, O'Connor K (2006). Seven practices for effective learning. Best of Educational Leadership 2005-2006.

[18] Clark I (2012). Formative assessment: assessment is for self-regulated learning. Educ Psychol Rev.

